# The association between weight-waist-adjustment index and serum folate in US adults: NHANES 2013 to 2018

**DOI:** 10.1097/MD.0000000000042313

**Published:** 2025-05-09

**Authors:** Zilin Zhou, Qi Zhao

**Affiliations:** a Department of Acupuncture, First Teaching Hospital of Tianjin University of Traditional Chinese Medicine, Tianjin, China; b National Clinical Research Center for Chinese Medicine Acupuncture and Moxibustion, Tianjin, China.

**Keywords:** cross-sectional study, NHANES, obesity, serum folate, weight-adjusted-waist index

## Abstract

Obesity is closely related to human metabolism and a variety of diseases. The association between weight-waist-adjustment index (WWI, a new index for obesity) and serum folate has not been sufficiently explored. Data from the National Health and Nutrition Examination Survey (2013–2018) was used to explore the correlation between WWI and serum folate. 12,757 adult participants were included in our study. In order to discern the relationship, we conducted weighted multiple linear regression analysis, generalized weighted smooth curve fitting and threshold effect analyses. Additionally, we executed subgroup analysis and interaction tests. There was a negative correlation between WWI and serum folate. In subgroup analysis, the relationship between WWI and serum folate were more pronounced among females, the elderly (65–80 years), nonsmokers, and those with hypertension or stroke. Furthermore, a nonlinear association between WWI and serum folate was found using smooth curve fitting (likelihood ratio = 0.014), with a threshold identified at WWI of 7.42. We discovered a stronger association between WWI and serum folate than other obesity markers including body mass index and waist circumference. Our study could help obese people predict serum folate and manage their nutrition well.

## 1. Introduction

Obesity, influenced by an intricate interplay of environmental, genetic, metabolic, and behavioral factors, has emerged as a critical public health concern.^[1]^ Over the last 10 years, the prevalence of obesity in the US has increased dramatically, reaching epidemic proportions.^[2]^ The most recent predictions indicate that by 2030, 57.8% of the global population will become obese or overweight.^[3]^ The alarming rise in obesity has been closely associated with an increased risk of developing various chronic health conditions, such as type 2 diabetes, high blood pressure, heart disease, stroke, and cancer.^[4,5]^ Additionally, obesity has been recognized as a major factor contributing to the prevalence of depression and other mental health disorders.^[6]^ Consequently, the health challenges posed by obesity have become a focal point, demanding comprehensive research and effective interventions.

Folate, a crucial water-soluble vitamin, is mostly obtained from fruits, leafy green vegetables, and liver.^[7]^ Folic acid and its derivatives play pivotal roles in numerous vital biochemical reactions and metabolic processes.^[8]^ In clinical practice, when assessing the risk of folate status among individuals in the Americas, a commonly used threshold value is a serum folate concentration below 7 nmol/L (3μg/L), which is considered indicative of serum folate deficiency. Conversely, concentrations above this threshold are regarded as indicative of no deficiency.^[9]^ A deficiency in folate has been associated with various diseases. For instance, insufficient folate can exacerbate inflammatory conditions and increase the likelihood of renal fibrosi.^[10]^ Reduced folate levels can adversely impact cognitive function through systemic inflammatory responses.^[11]^ Furthermore, insufficient levels of serum folate can lead to elevated homocysteine levels, thereby increasing the likelihood of developing ischemic heart disease, stroke, and depression.^[12–14]^

It has been reported that the prevalence of folate deficiency is significantly higher in obese populations, indicating a close association between obesity and folate levels. However, the specific underlying mechanisms of this association have not been fully elucidated.^[15]^ Obesity is characterized by various hormonal changes, inflammatory responses, and alterations in endothelial function, all of which set off intricate complex biological processes that increase the risk of disease.^[16]^ Research has shown that obesity is a chronic low-grade inflammatory condition, in which the occurrence of inflammatory responses influences alterations in hematological parameters and regulates the expression levels of various elements in the serum.^[17]^ Relevant research has confirmed that individuals who are overweight or obese tend to exhibit lower serum folate levels, potentially due to the impact of excessive fat accumulation on metabolic reactions within the body.^[18]^ In obese individuals, adipose tissue secretes a large amount of pro-inflammatory adipokines, which, through direct or indirect actions on immune cells, activate inflammasomes, thereby triggering local and even systemic inflammatory responses, leading to severe metabolic dysfunction.^[19]^ Folate, as an essential nutrient, its deficiency state is closely associated with pathological processes such as impaired vascular endothelial function and dyslipidemia, and constitutes an important component of the pathogenesis of metabolic syndrome.^[20]^ Therefore, obesity may be closely related to serum folate levels in the body through its impact on metabolism, although the exact relationship between the 2 requires further investigation.

An increasing number of studies have confirmed the potential association between obesity indices and serum folate. For instance, a study from Canada observed a negative association between serum folate levels and body mass index (BMI) among pregnant women.^[21]^ Similarly, research from China found that higher serum folate levels were correlated with a lower risk of obesity among children and adolescents.^[22]^ Additional research has revealed that serum folate levels are generally lower in the obese population of the United States.^[23]^ Across these studies, BMI has been frequently used as the standard for defining overweight and obesity. Although the World Health Organization’s has recommended using BMI as the principal standard for assessing overweight and obesity,^[24]^ BMI still has its limitations and controversies, as it fails to accurately reflect central obesity and visceral fat distribution.^[25]^ Conversely, waist circumference (WC) is more closely correlated with visceral obesity, and some researchers argue that combining BMI with WC can more effectively predict the risk of obesity-related diseases.^[26]^ Moreover, the weight-adjusted-waist index (WWI), a weight-standardized index derived from WC, has been proposed as a novel obesity indicator. This index aims to evaluate its association with obesity-related diseases, cardiovascular mortality, and overall mortality, providing a novel approach to measuring central obesity.^[27]^ However, up to now, no study has examined the association between WWI and serum folate levels. In this study, we utilized data from the 2013 to 2018 National Health and Nutrition Examination Survey (NHANES) to comprehensively explore the association between WWI and serum folate.

## 2. Materials and methods

### 2.1. Data source

The NHANES is a nationwide survey administered by the Centers for Disease Control and Prevention (CDC) of the United States. As a nationally representative cross-sectional survey, its major goal is to assess the health and nutritional status of adults and children in the United States.^[28]^ Using a sophisticated multistage probability sampling design, a representative sample of the US population is chosen for the assessment. Participants undergo a range of evaluations, which encompassed physical examinations, health and nutrition questionnaires, as well as laboratory tests.

The research has obtained formal approval from the Ethics Review Committee of the National Center for Health Statistics (NCHS). All individuals involved provided their inform consent. All research design protocols and associated data from the NHANES are publicly accessible at https://www.cdc.gov/nchs/nhanes/. This study rigorously adhered to the guidelines for data usage, ensuring that all data were exclusively employed for scientific statistical analysis purposes.^[29]^

### 2.2. Study population

For this research, data were drawn from 3 consecutive NHANES cycles (2013–2014, 2015–2016, and 2017–2018), encompassing a total of 29,400 individuals. We excluded participants without serum folate (N = 8330), BMI (N = 637), and WC (N = 589). Subsequently, we excluded the participants under 20 years of age (N = 6993). Lastly, we excluded participants without other covariates, including coronary heart disease, hypertension, diabetes, and stroke (N = 94).

**Figure 1.**
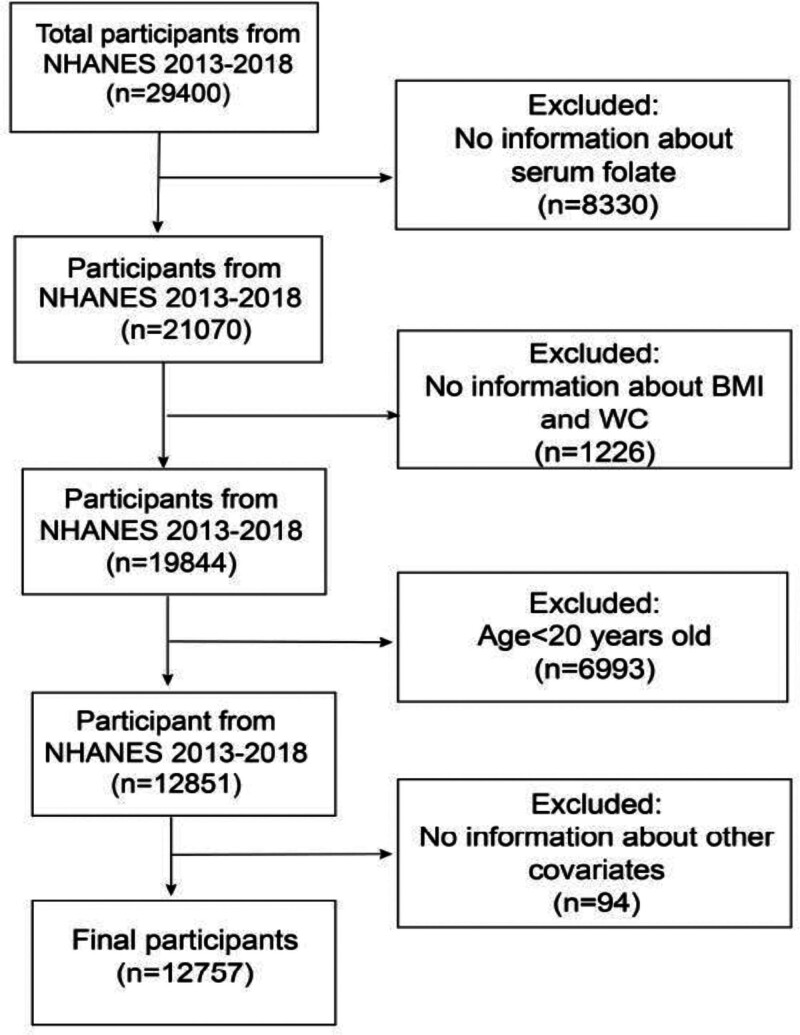
Flowchart depicting the participants’ selection.

Ultimately, a total of 12,757 people were incorporated into the analysis. Figure 1 presented the detailed flowchart illustrating the inclusion and exclusion criteria used in our study.

### 2.3. Obesity indicators

WWI (cm/√kg) is computed by dividing WC (cm) by the square root of body weight (kg).^[27]^ Higher WWI indicates higher levels of obesity. Anthropometric measurements were conducted by a team of skilled health professionals from the Mobile Examination Center (MEC) following standardized protocols. These protocols, along with quality control procedures, are thoroughly documented in the NHANES. WWI was treated as continuous variable in our analysis and grouped according to quartiles. Additionally, we chose BMI and WC as our alternative obesity indicators. BMI is calculated by dividing the individual’s weight in kilograms by the square of their height in meters. The cutoff values chosen to define overweight (25 < BMI < 30 kg/m^2^) and obesity (BMI ≥ 30 kg/m^2^) are internationally recognized. Abdominal obesity is defined as a WC of ≥102 centimeters for men and ≥88 centimeters for women.^[30]^

### 2.4. Serum folate levels

The outcome variable was serum folate. In NHANES laboratories, the measurement of serum folate was conducted using the isotope dilution high-performance liquid chromatography-tandem mass spectrometry method. To achieve precise quantification, this method involved incorporating a number of folate derivatives, such as 5-methyl-tetrahydrofolate, pteroylglutamic acid, 5-formyl-tetrahydrofolate, tetrahydrofolate, and 5,10-methenyl-tetrahydrofolate. It was known for its high accuracy and specificity. Overnight fasting prior to specimen collection was recommended.^[31]^

### 2.5. Covariates

The covariates encompassed demographic, lifestyle, health status, and laboratory examination variables. All the data were collected through household interviews and questionnaires administered by trained interviewers during household interviews and at the MEC. Specifically, demographic data, including age, sex, race (including Mexican American, Other Hispanic, Non-Hispanic White, Non-Hispanic Black, and others), educational level (categorized as <9 years, 9 to 12 years, or over 12 years), marital status (married/cohabitating or solitary), and family income levels (classified as low, medium, and high based on the poverty income ratio thresholds defined by the U.S. government). Information on lifestyle habits was obtained through questionnaire-based surveys, focusing on smoking status (current, former, or nonsmoker) and alcohol intake (drinker or nondrinker). Additionally, health status data was also collected through self-reported questionnaire surveys, including medical histories of hypertension, diabetes mellitus (DM), coronary heart disease, stroke, and sleep disorders. All participants were asked if they had been diagnosed with any of these conditions by their doctor or other healthcare professionals. Laboratory testing included the measurement of high-density lipoprotein cholesterol (HDL-C) levels, cholesterol, uric acid, and the ratio of albumin to creatinine. The detailed measurement procedures for these variables are publicly accessible at www.cdc.gov/nchs/nhanes/.

### 2.6. Statistical analysis

The sampling weights, stratification, and clustering provided in the NHANES study were employed to address the complex sampling design that was used for the selection of a representative sample of non-institutionalized Americans, thus reflecting the diverse demographic characteristics of the target population. The data analysis adhered to the NHANES guidelines. Given the use of data from 3 consecutive cycles, appropriate weighting adjustments were applied in the computation of statistics.

Categorical variables were expressed as percentages, and weighted Chi-square tests were conducted to assess the disparities among groups. Continuous variables were presented as means ± standard deviations, and weighted Student *t* tests were used to evaluate differences between groups. In compliance with guidelines, a weighted multiple linear regression analysis was conducted to investigate the potential association between WWI and serum folate. The linear association between participants’ WWI and serum folate were assessed across 3 models. To evaluate the independent effects of predictors on the outcome, 3 models were generated, each with a different set of adjusted predictors. Model 1 was unadjusted. Model 2 incorporated sex, age, and race as common demographic variables. Model 3 adjusted for all covariates (gender, age, race, education level, family income, marital status, coronary heart disease, stroke, sleep disorders, hypertension, DM, drink, smoking status, HDL-C, cholesterol, uric acid, and albumin/creatinine), encompassing both demographic factors and additional variables previously identified as relevant to serum folate or obesity.

Subgroup analysis was carried out better understand the relationship between WWI and serum folate. Subsequently, a generalized weighted smoothing spline curve fitting method was conducted to explore the potential nonlinear association between obesity and serum folate or not. To confirm the results of this research and identify significant inflection points, threshold effect analysis was also used. Missing values for covariates were handled by imputing the median due to minimal covariate missingness and the continuous nature of the variables. For this survey, R software (https://www.R-project.org) and Empower software (https://www.empowerstats.com) were used for data processing and statistical analysis. *P* < .05 was regarded as statistical significance.

## 3. Results

### 3.1. Characteristics of study population

The baseline characteristics of the study population are presented in Table 1. There were 12,757 participants in all, with a mean age of 46.90 ± 16.82 years. Of them, 54.08% were females and 45.92% were males. In summary, the mean serum folate level among all participants was 44.291 ± 36.643, with a mean WWI of 8.097. Additionally, the serum folate level decreased as the WWI increased. Based on their WWI values, participants were divided into 4 weighted quartiles: Q1 (<7.02 cm/√kg), Q2 (7.02–7.92cm/√kg), Q3 (7.92–9.01cm/√kg), and Q4 (>9.01cm/√kg). Compared to participants in the lowest quartile of WWI, those in the highest quartile were more likely to be middle-aged or elderly, male, have a higher educational level, be nonsmokers, and have higher levels of uric acid, albumin/creatinine, BMI, and WC. With the exception of the coronary heart disease, baseline characteristics showed statistically significant differences between the WWI quartiles and all covariate factors (*P* < .05).

**Table 1:** General characteristics of participants (n = 12757) stratified by weight-waist-adjustment index (quartiles 1–4, cm/√kg) in the NHANES 2013 to 2018.

Characteristic	Total (n = 12757)	Q1 (<7.02) n = 3189	Q2 (7.02–7.92) n = 3187	Q3 (7.92–9.01) n = 3188	Q4 (>9.01) n = 3193	*P*-value
Age (yr) mean (SD)	46.904 ± 16.816	48.405 ± 19.050	47.468 ± 17.381	47.069 ± 16.150	45.131 ± 14.835	<.001
20 to 39	39.764	39.731	39.379	38.372	41.384	
40 to 64	43.836	36.948	41.585	46.678	48.411	
65 to 80	16.401	23.321	19.036	14.950	10.205	
Gender, n						
Man	45.916	14.924	37.672	57.305	66.179	<.001
Woman	54.084	85.076	62.328	42.695	33.821	
Race, n (%)						
Mexican American	9.090	9.176	10.398	8.781	8.198	<.001
Other Hispanic	6.218	7.904	6.716	5.575	5.097	
Non-Hispanic White	64.628	59.652	63.422	67.253	67.056	
Non-Hispanic Black	10.905	7.182	10.010	11.455	14.003	
Other Race	9.158	16.086	9.454	6.936	5.645	
Education level, years, n (%)						
<9	4.558	6.485	5.784	4.296	2.285	<.001
9 to 12	8.915	10.328	8.667	9.715	7.318	
>12	86.527	83.187	85.549	85.988	90.396	
Martial status, n (%)						
Married or living with a partner	63.885	58.225	62.783	65.971	67.239	<.001
Living alone	36.115	41.775	37.217	34.029	32.761	
Family income, n (%)	2.930 ± 1.601	2.765 ± 1.601	2.881 ± 1.601	3.005 ± 1.599	3.027 ± 1.593	<.001
Low income	20.210	23.021	21.108	18.937	18.465	
Medium income	40.459	42.118	40.696	39.682	39.700	
High income	39.331	34.861	38.196	41.382	41.835	
Drink (had at least 12 alcoholic drinks/1 yr?), n (%)						
Yes	10.242	14.220	10.744	9.275	7.660	<.001
No	29.669	31.513	29.654	27.461	30.288	
Missing or unkown	60.089	54.267	59.602	63.264	62.052	
Smoke, n (%)						
No	57.077	64.003	57.275	56.223	52.399	<.001
Used to	24.307	17.855	21.832	25.847	29.927	
Yes	18.616	18.142	20.894	17.930	17.674	
Hypertension, n (%)						
Yes	32.148	24.810	28.562	32.453	40.513	<.001
No	67.852	75.190	71.438	67.547	59.487	
DM, n (%)						
Yes	9.969	5.388	7.845	11.577	13.802	<.001
No	87.634	92.813	90.103	86.158	82.933	
Borderline	2.397	1.799	2.053	2.264	3.266	
CHD, n (%)						
Yes	3.409	2.936	3.758	3.535	3.359	.362
No	96.591	97.064	96.242	96.465	96.641	
Stroke, n (%)						
Yes	2.492	3.080	2.894	2.163	2.004	.012
No	97.508	96.920	97.106	97.837	97.996	
Sleep disorder, n (%)						
Yes	29.719	27.775	27.721	27.606	34.827	<.001
No	70.281	72.225	72.279	72.394	65.173	
HDL-C (mg/dL)	54.362 ± 17.023	64.073 ± 19.425	57.284 ± 16.294	52.057 ± 14.887	46.571 ± 12.686	<.001
Uric acid (μmol/L)	317.697 ± 82.442	272.600 ± 70.307	298.697 ± 75.010	328.911 ± 75.729	358.013 ± 81.077	<.001
Albumin/creatinine	33.402 ± 293.989	38.719 ± 410.764	24.525 ± 153.438	24.242 ± 139.851	45.224 ± 370.949	.005
Cholesterol	4.961 ± 1.076	4.961 ± 1.049	5.000 ± 1.090	4.982 ± 1.051	4.910 ± 1.106	.004
Serum folate	44.291 ± 36.643	49.166 ± 33.690	44.969 ± 25.825	43.047 ± 24.530	41.129 ± 52.155	<.001
BMI (kg/m^2^)	29.361 ± 6.995	22.836 ± 3.163	26.512 ± 3.552	29.788 ± 4.238	36.369 ± 7.003	<.001
WC (cm)	100.085 ± 16.866	83.768 ± 9.671	93.124 ± 9.816	101.756 ± 10.594	116.919 ± 14.715	<.001

BMI = body mass index, CHD = coronary heart disease, DM = diabetes mellitus, HDL-C = high-density lipoprotein cholesterol, WC = waist circumference.

Table 1 showed the overall traits of the participants (n = 12757) categorized by their weight-waist-adjustment index (quartiles 1–4，cm/√kg) in the NHANES 2013 to 2018.

### 3.2. Relationship between WWI and serum folate

We employed weighted multiple linear regression analysis to investigate the association between WWI and serum folate. Our findings indicated that higher WWI was significantly associated with decreased serum folate levels. A negative correlation between WWI and serum folate was detected in the fully adjusted model (β = −1.378, 95% CI: −1.857 to −0.898). Moreover, as WWI increased, the negative association with serum folate became increasingly significant (*P* for trend < .001). Specifically, in the fourth quartile of WWI, the effect on serum folate was (β = −5.188, 95% CI: −7.450 to −3.216). In our analysis, we observed a notably stronger correlation between WWI and serum folate, both in terms of continuous and categorical factors, compared to BMI and WC. According to these findings, WWI provides a more comprehensive understanding of the relationship between obesity and serum folate levels. All results were shown in Table 2.

**Table 2:** The association between BMI (kg/m^2^), WC(cm) and WWI (cm/√kg)with serum folate (nmol/L).

Exposure	Model 1		Model 2		Model 3	
	[β(95% CI)]	*P*-value	[β(95% CI)]	*P*-value	[β(95% CI)]	*P*-value
BMI (kg/m^2^) continuous	−0.225 (−0.316 to −0.134)	.963	−0.254 (−0.344 to −0.165)	.160	−0.321 (−0.424 to −0.218)	.081
Quartiles of BMI (kg/m^2^)						
≤25	0		0		0	
25 to 29.9	0.038 (−1.593 to 1.670)	.963	−1.159 (−2.776 to 0.459)	.160	−1.489 (−3.166 to 0.187)	.082
≥30	−3.247 (−4.806 to −1.689)	<.001	−4.243 (−5.787 to −2.699)	<.001	−5.145 (−6.898 to −3.393)	<.001
*P* for trend	<.001		<.001		<.001	
WC continuous	−0.025 (−0.063 to 0.012)	.189	−0.087 (−0.125 to −0.049)	<.001	−0.106 (−0.150 to −0.062)	<.001
Quartiles of WC (cm)						
Normal	0		0		0	
Obese	1.487 (0.192 to 2.782)	.024	−2.704 (−4.033 to −1.374)	<.001	−2.854 (−4.309 to −1.399)	<.001
*P* for trend	<.001		<.001		<.001	
WWI Continuous	−2.027 (−2.442 to −1.612)	<.001	−1.137 (−1.580 to −0.695)	<.001	−1.450 (−1.946 to −0.954)	<.001
Quartiles of WWI (cm/√kg)						
Q1	0		0		0	
Q2	−4.198 (−6.075 to −2.320)	<.001	−2.838 (−4.706 to −0.969)	.003	−2.852 (−4.735 to −0.969)	.003
Q3	−6.119 (−7.965 to −4.273)	<.001	−3.895 (−5.807 to −1.984)	<.001	−4.305 (−6.367 to −2.405)	<.001
Q4	−8.037 (−9.846 to −6.229)	<.001	−4.553 (−6.484 to −2.622)	<.001	−5.188 (−7.450 to −3.216)	<.001
*P* for trend	<.001		<.001		<.001	

Model 1: No covariates were adjusted.

Model 2: Age, gender and race were adjusted.

Model 3: Gender, age, race, education level, family income, marital status, coronary heart disease, stroke, sleep disorders, hypertension, DM, drink, smoking status, HDL-C, cholesterol, uric acid, and albumin/creatinine were adjusted.

BMI = body mass index, WWI = weight-waist-adjustment index.

### 3.3. Subgroup regression analysis between WWI and serum folate

To validate the robustness of the negative correlation between WWI and serum folate levels across different cohorts, we conducted a comprehensive subgroup analysis and interaction tests. Stratification was performed based on various factors including gender, age, race, education level, smoking status, diabetes, hypertension, coronary heart disease, stroke, and sleep disorders, revealing potential demographic disparities.

The study findings indicated significant interactions (*P* for interaction < .05) between WWI and serum folate levels across gender, age, smoking, hypertension, and stroke. In addition, stratified analyses revealed a stronger negative association between obesity and serum folate. For instance, in subgroup analyses stratified by hypertension prevalence status, the absolute effect size was significantly higher in hypertensive patient [WWI: (β = −3.599, 95% CI: −4.193 to −3.005)]. In contrast, education level, diabetes, coronary heart disease, and sleep disorders did not significantly change this negative association (*P* for interaction > .05). Table 3 presented the subgroup analysis results, demonstrating a dose-response connection between WWI and serum folate.

**Table 3:** Subgroup regression analysis between WWI (cm/√kg) with serum folate (nmol/L).

Characteristic	Serum folate (OR [95%CI])	*P* for interaction
Stratified by gender		
Male	−0.920 (−1.516 to −0.325)	<.001
Female	−3.238 (−3.761 to −2.715)	
Stratified by age (yr)		
20 to 39	−1.176 (−1.748 to −0.605)	<.001
40 to 64	−2.179 (−2.747 to −1.612)	
65 to 80	−3.419 (−4.374 to −2.464)	
Stratified by race, n (%)		
Mexican American	−2.570 (−3.612 to −1.529)	<.001
Other Hispanic	−3.325 (−4.572 to −2.078)	
Non-Hispanic White	−2.482 (−3.092 to −1.871)	
Non-Hispanic Black	−1.219 (−2.020 to −0.419)	
Other Race	−4.103 (−5.114 to −3.093)	
Stratified by education (yr)		
<9	−3.605 (−5.064 to −2.146)	0.225
9 to 12	−3.051 (−4.175 to −1.927)	
>12	−2.444 (−2.860 to −2.027)	
Stratified by smoke, n (%)		
No	−3.263 (−3.747 to −2.779)	<.001
Used to	−2.026 (−2.831 to −1.221)	
Yes	−1.299 (−2.158 to −0.440)	
Stratified by CHD		
Yes	−4.079 (−6.087 to −2.070	.135
No	−2.518 (−2.899 to −2.138)	
Stratified by blood pressure		
Yes	−3.599 (−4.193 to −3.005)	.002
No	−2.405 (−2.890 to −1.920)	
Stratified by DM		
Yes	−3.188 (−4.171 to −2.205)	.534
No	−2.664 (−3.078 to −2.250)	
borderline	−3.373 (−5.495 to −1.250)	
Stratified by stroke		
Yes	−7.415 (−9.412 to −5.419)	<.001
No	−2.362 (−2.743 to −1.981)	
Stratified by sleep disorder		
Yes	−1.771 (−2.438 to −1.105)	<.001
No	−3.044 (−3.499 to −2.589)	

CHD = coronary heart disease, DM = diabetes mellitus, WWI = weight-waist-adjustment index.

### 3.4. Nonlinear relationship of BMI, WC and WWI with serum folate

**Figure 2.**
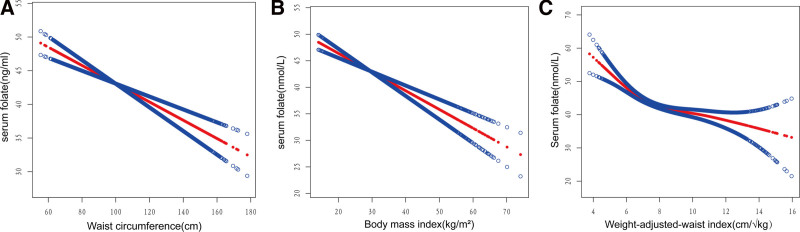
Smooth curve fitting for WWI, BMI, and WC with serum folate.

Using threshold effect analysis and smooth curve fitting, we were able to further evaluate any nonlinear relationships that might exist between the independent factors and serum folate levels. After adjustment for all relevant covariates, our analysis revealed a nonlinear correlation between WWI and serum folate levels, which exhibited statistically significant differences (likelihood ratio [LLR] = 0.014) in Figure 2. Subsequently, threshold effect analysis revealed a saturation point of 7.42 cm/√kg for the WWI-serum folate relationship among all participants. When BMI and WC were used as independent variables, Figure 2 indicated linear relationships between BMI, WC, and serum folate levels. Subsequent threshold effect analysis confirmed these linear relationships (LLR = 0.183 for BMI and LLR = 0.004 for WC). The detailed findings of the threshold effect analysis are summarized in Table 4.

**Table 4:** Threshold effect analysis for association of WWI (cm/√kg)、WC (cm) and BMI (kg/m^2^) with serum folate (nmol/L).

Exposures	BMI	WC	WWI
Linear effect model β, (95% CI)	−0.321 (−0.424 to −0.218) <0.001	−0.106 (−0.150 to −0.062) <0.001	−1.450 (−1.946 to −0.954) <0.001
Nonlinear mode			
Infection point (K)	20.2	130.1	7.42
β (95% CI) (<K)	0.802 (−0.857 to 2.462) 0.221	−0.143 (−0.194 to −0.092) <0.001	−3.186 (−4.663 to −1.710) <0.001
β (95% CI) (≥K)	−0.338 (−0.444 to −0.232) <0.001	0.218 (−0.007 to 0.443) 0.058	−1.017 (−1.622 to −0.412) 0.001
LLR	0.183	0.004	0.014

Adjusted for gender, age, race, education level, family income, marital status, CHD, stroke, sleep disorders, hypertension, DM, drink, smoking status, HDL-C, cholesterol, uric acid, and albumin/creatinine.

BMI = body mass index, CHD = coronary heart disease, DM = diabetes mellitus, HDL-C = high-density lipoprotein cholesterol, LLR = likelihood ratio, WC = waist circumference, WWI = weight-waist-adjustment index.

## 4. Discussion

This survey investigated the association between weight-adjusted-waist index (WWI) and serum folate levels. Our study uncovered a significant negative correlation between obesity indicators and serum folate, indicating that individuals with higher WWI tend to have lower serum folate concentrations. Notably, WWI emerged as a novel obesity indicator, offering a more precise representation of the negative correlation compared to traditional indicators. These findings indicate that WWI has the potential to serve as a more precise predictor of serum folate, potentially offering valuable insights for future research and clinical practice.

Based on our current understanding, our study represents the first comprehensive cross-sectional exploration of the association between WWI and serum folate levels. This investigation sheds light on the correlation between higher WWI and decreased serum folate levels. Globally, BMI and WC have become accepted standards for defining obesity.^[32,33]^ There is a consistent negative correlation between BMI, WC and serum folate, as shown by several studies. For instance, a Korean study involving 6394 adults aged 19 to 80 used complicated sample logistic regression analysis to investigate the association between serum folate levels, obesity indicators and inflammatory markers, confirming a significant negative correlation between BMI, WC and serum folate.^[34]^ Another cohort study of 421 healthy individuals, aged 20 to 40, provided further evidence of a linkage between low serum folate levels and weight gain, as well as central obesity, as indicated by higher BMI, larger WC, and increased adiposity.^[35]^ Remarkably, these findings align with our study results, although they focused solely on BMI and WC, without investigating the relationship between WWI and serum folate. Notably, in comparison to traditional indicators such as BMI and WC, WWI is considered a reliable tool for measuring obesity, as it better reflects various changes in body composition and possesses a broader range of applicability.^[36]^ In diverse research domains, WWI has garnered considerable attention owing to its profound association with the heightened prevalence of hypertension, stroke and cardiovascular diseases.^[37–39]^ Concurrently, when compared to other obesity indices, WWI exhibits a more pronounced correlation. Specifically, research has indicated that, in contrast to BMI and WC, WWI displays a stronger positive correlation with diabetes, thereby enabling more effective prediction of diabetes risk.^[40]^ Furthermore, another study has also found that there is a significant positive correlation between WWI and urinary albumin excretion, and its correlation surpassing that of other obesity indicators such as BMI and WC.^[41]^ These findings are consonant with our results. In our analysis, WWI exhibited a nonlinear association with serum folate in both unadjusted and fully adjusted models, with significantly lower serum folate levels in the fourth quartile of WWI compared to the first quartile (β = −5.188, 95% CI: −7.450 to−3.216), indicating a significant negative impact of obesity on serum folate. Moreover, our study revealed that the correlation between WWI and serum folate levels was considerably more prominent in comparison to that of BMI and WC (WWI: β = −1.450; BMI: β = −0.321; WC: β = −0.106), which indicates that WWI demonstrates superior predictive capability for serum folate levels. Of particular note, our analysis uncovered an L-shaped relationship between WWI and serum folate, with a turning point at 7.42 cm/√kg.

Additionally, our in-depth subgroup analysis and interaction tests uncovered a continuous and statistically significant negative association between WWI and serum folate across various subgroups, including gender, age, smoking status, hypertension, and stroke. Specifically, WWI was more strongly correlated with serum folate in females, elderly individuals (65–80 years), nonsmokers, and those with hypertension and stroke. When BMI was employed as the obesity criterion, females showed a considerably higher incidence of obesity compared to males across all sociodemographic categories, according to a study looking at the prevalence of overweight and obesity in 195 countries.^[42]^ Another study confirmed that obese women displayed a reduced total serum folate response (0–10 hours) compared to women of normal weight, suggesting that obesity has a major impact on the body’s distribution of serum folate.^[43]^ This implied that women, particularly those with obesity, may be at a higher risk of experiencing low serum folate levels. Regarding the relationship between age and serum folate, we observed that older participants displayed more significant effect sizes. Previous research has shown that the American population’s serum folate intake varies significantly by age, with those over 65 having a higher risk of serum folate insufficiency.^[44]^ Researchers studying healthy Greeks found that nonsmokers’ serum folate levels were 13% higher than smokers’.^[45]^ Meanwhile, another study identified a positive correlation between smoking and central obesity, implying that smoking may foster the accumulation of adipose tissue.^[46]^ This emphasized how smoking had a significant impact on the association between obesity and serum folate. Additionally, several studies have shown a connection between serum folate, cardiovascular diseases and stroke. Hypertensive patients with lower folate levels demonstrated a significantly increased risk of stroke.^[47,48]^ Moreover, a robust correlation has been identified between the WWI and the incidence of hypertension in the elderly, suggesting that WWI could be a unique biomarker for early identification of hypertension risk in older populations.^[49]^ Therefore, close attention should be paid to changes in obesity indices, particularly WWI, in these specific populations.

The above relationships can be explained through physiological and pathological pathways. Obesity is a chronic low-grade state of inflammation, characterized by alterations in inflammatory cell populations and the subsequent tissue injury, which ultimately results in a significant elevation of inflammatory markers and cells circulating in the bloodstream.^[50]^ This inflammatory response process, known as metabolic inflammation, is strongly linked to lifestyle factors, particularly the quality of dietary intake and physical activity levels.^[51]^ Excess adipose tissue, acting as a metabolic and endocrine organ, can disrupt the body’s metabolic balance and lead to vitamin deficiencies, thereby exacerbating pathological conditions.^[52]^ Research indicates that abdominal obesity is primarily attributed to the excessive accumulation of metabolically active visceral fat surrounding the organs, which is often accompanied by the expansion of subcutaneous fat and an increase in metabolic risks.^[53]^ Compared to subcutaneous adipose tissue, visceral fat contains a higher abundance of inflammatory and immune cells.^[54]^ Excess visceral fat leads to endoplasmic reticulum stress, increased expression of inflammatory regulatory factors, and the activation of inflammatory signaling pathways, thereby exacerbating metabolic disorders and increasing the risk of developing metabolic syndrome.^[55]^ Dysregulation of folate metabolism may involve various metabolic changes, including metabolic syndrome,^[56]^ fatty liver,^[57]^ and dyslipidemia.^[58]^ Additionally, folate deficiency may promote lipid accumulation in fat cells and leptin production, potentially serving as an underlying risk factor for obesity.^[59]^ Therefore, we hypothesize that excessive accumulation of visceral fat may disrupt metabolic homeostasis and affect folate levels in the body. WWI demonstrates a positive correlation with abdominal fat area and a negative correlation with muscle mass, thereby enabling a more precise assessment of adverse metabolic characteristics in individuals.^[36]^ Consequently, as an effective tool for assessing central obesity, WWI not only reflects obesity status more accurately but may also be more closely associated with folate metabolism. Since the WWI is derived from a comprehensive calculation involving WC and weight, in clinical practice, measuring WC and weight, in addition to BMI, can be more helpful in identifying and managing obese individuals who are at risk of folate deficiency. Adopting healthy lifestyles, such as scientifically controlled dietary intake and increased physical activity, can help reduce WC and body weight, decrease the accumulation of visceral fat, and thereby prevent the occurrence of obesity and folate deficiency-related diseases.

While acknowledging the limitations of our research, it is crucial to highlight the various obstacles that have been encountered. First of all, due to the cross-sectional nature of design, we are constrained from establishing a causal link between obesity indices and serum folate. Secondly, even though we have accounted for various significant factors that could affect the results, the potential impact of other unknown variables cannot be fully eliminated, thus demanding a cautious interpretation of the results. Furthermore, due to the relatively small sample size, we were unable to perform a weighted binary logistic regression analysis with the dependent variable based on the criteria for folate deficiency, in order to identify factors associated with obesity and folate deficiency. Lastly, our data is confined to a single country, which raises questions about the generalizability of our findings. However, despite these limitations, our study possesses several noteworthy strengths. First and foremost, our research is grounded in data from the nationally representative NHANES, which utilizes sample weights to accurately reflect the overall US population. Additionally, we performed subgroup analyses with the large sample size to explore the robustness of the association between obesity and serum folate in a variety of population types. Notably, we also investigated the negative connection between WWI and serum folate, finding it to be more strongly correlated than BMI and WC.

## 5. Conclusions

Our study found a negative relationship between WWI and serum folate, with WWI demonstrating stronger predictive ability for serum folate compared to BMI and WC. This suggests that individuals with higher WWI values may exhibit lower serum folate levels. Identifying the association between WWI and folate offers opportunities for maintaining folate metabolism balance, reducing the risk of folate deficiency, and potentially lowering the incidence of metabolic diseases. At the same time, the negative correlation between obesity and serum folate, as measured by WWI, needed to be taken with caution especially among females, aged 65 to 80 years, nonsmokers, and those with hypertension and stroke. Future investigations, particularly longitudinal studies, are required to further explore the potential relationship between WWI and serum folate and to provide more precise and individualized solutions for the health management of obese populations.

## Acknowledgments

We would like to extend our thanks to the NHANES study staff and participants.

## Author contributions

**Conceptualization:** Zilin Zhou.

**Data curation:** Zilin Zhou.

**Formal analysis:** Zilin Zhou.

**Funding acquisition:** Qi Zhao.

**Investigation:** Zilin Zhou.

**Methodology:** Zilin Zhou.

**Software:** Zilin Zhou.

**Supervision:** Qi Zhao.

**Validation:** Qi Zhao.

**Visualization:** Qi Zhao.

**Writing – original draft:** Zilin Zhou.

**Writing – review & editing:** Qi Zhao.
